# Prolapsed Uterine Fibroid: Value of MRI in Case of Massive Bleeding

**DOI:** 10.5334/jbsr.3246

**Published:** 2023-08-30

**Authors:** Houda Azzouzi, Vasiliki Perlepe, Latifa Fellah

**Affiliations:** 1 Cliniques Universitaires Saint-Luc, BE

**Keywords:** Prolapsed uterine mass, MRI, Broccoli sign

## Abstract

**Teaching Point:** In case of acute bleeding caused by a mass located in the vagina, it may be difficult to assess the origin of the mass and determine whether it is benign or malignant; MRI is a useful tool for mass detection, diagnosis, and treatment decision.

## Case History

A 51-year-old female was referred to the gynecologic emergency department for abnormal acute vaginal bleeding. The patient lived abroad, and her medical history was unknown.

Physical examination revealed paleness, tachycardia, and hypotension (9/4 mmHg).

Gynecologic examination revealed a vaginal mass and active bleeding.

Transvaginal ultrasonography was performed in difficult conditions and differential diagnosis included prolapsed submucous uterine fibroid (PSUF), cervical, endometrial, vaginal or endometrial polyp.

**Figure 1:**
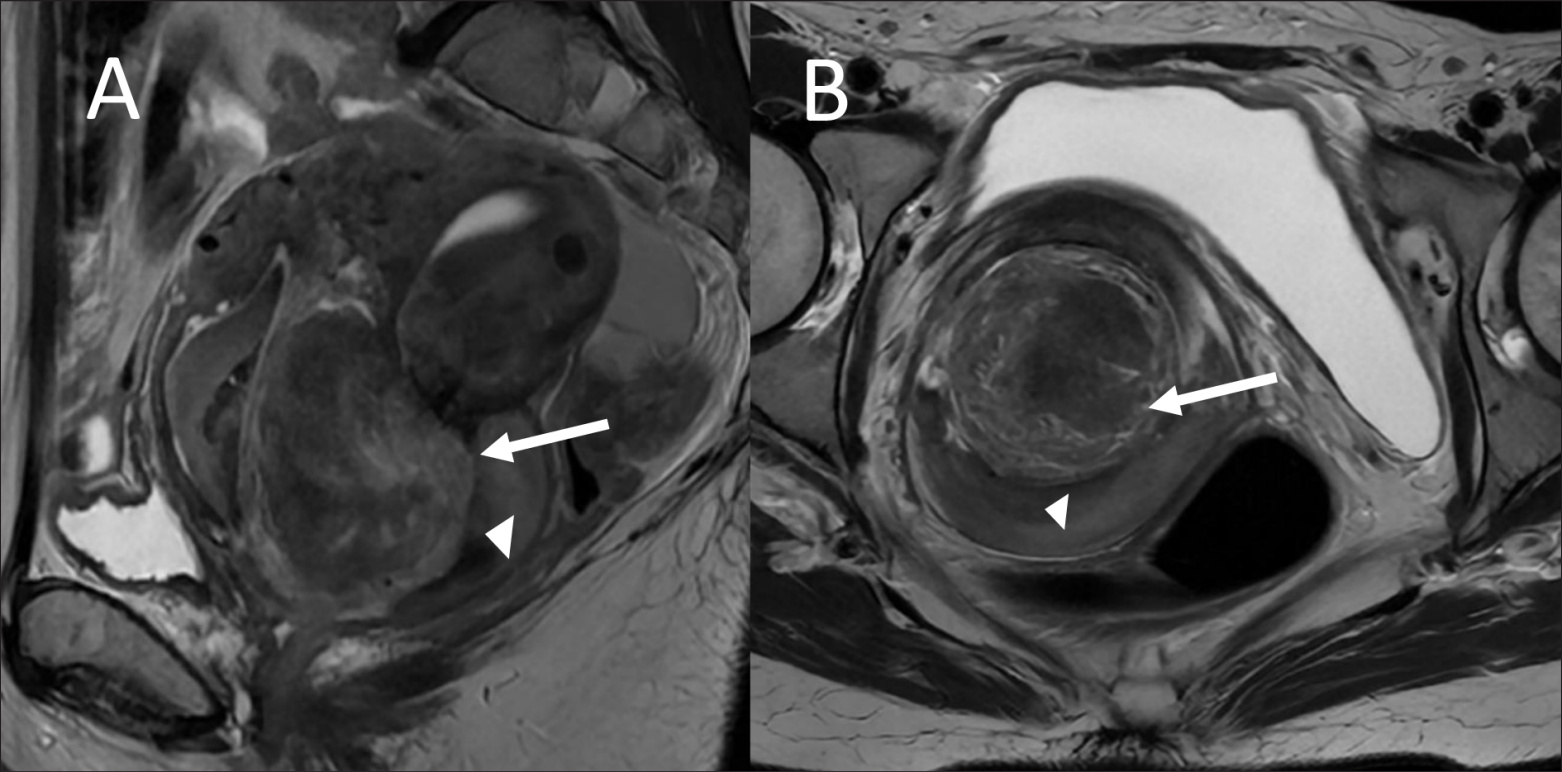


Biopsies were waived to avoid increasing bleeding, and emergency magnetic resonance imaging (MRI) was performed to determine adequate treatment. MRI sagittal (Figure 1A) and axial T2-weighted images (Figure 1B) showed a heterogeneous endometrial mass, measuring 7 cm, prolapsed in the cervical and vaginal canal from the uterine endometrium (white arrow) and vaginal bleeding (white head arrow).

**Figure 2:**
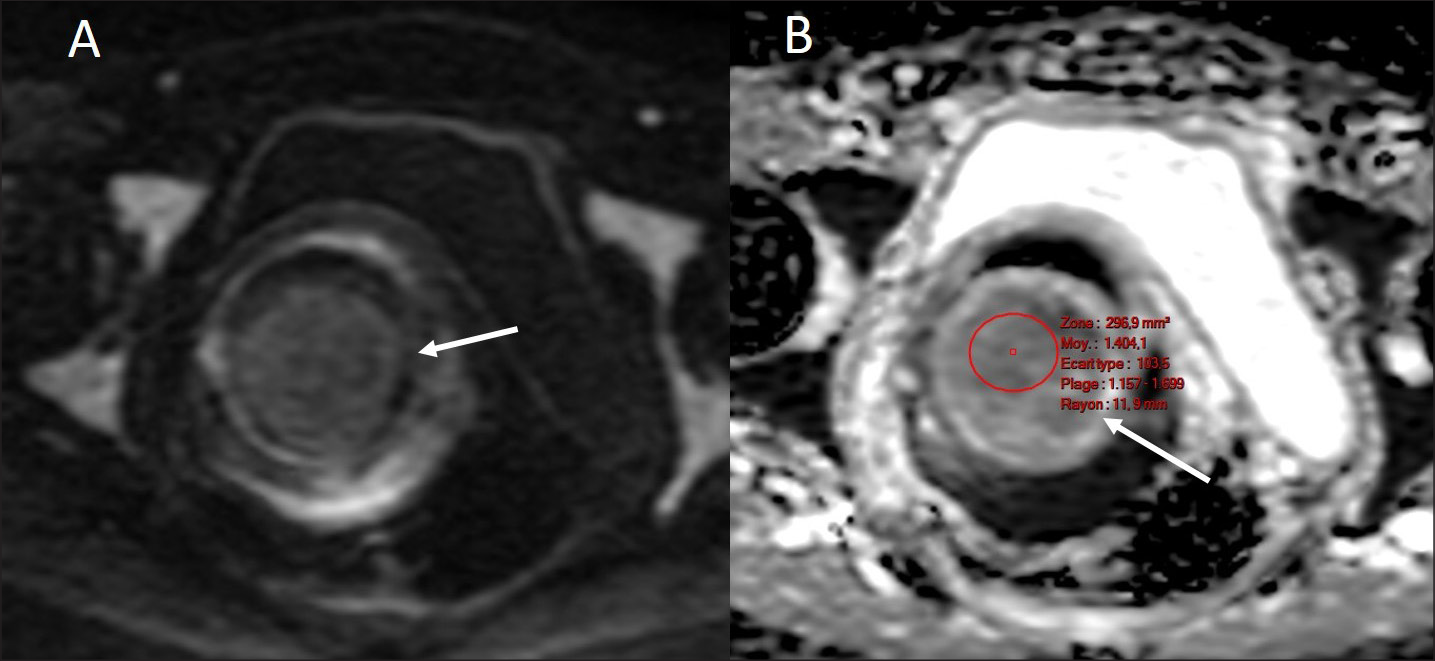


Axial diffusion-weighted image (Figure 2A) and apparent diffusion coefficient image (ADC) (Figure 2B) revealed a benign lesion with low signal intensity b_1000_ and normal ADC (1.4 10^-3^mm^2^/sec) (white arrow).

**Figure 3:**
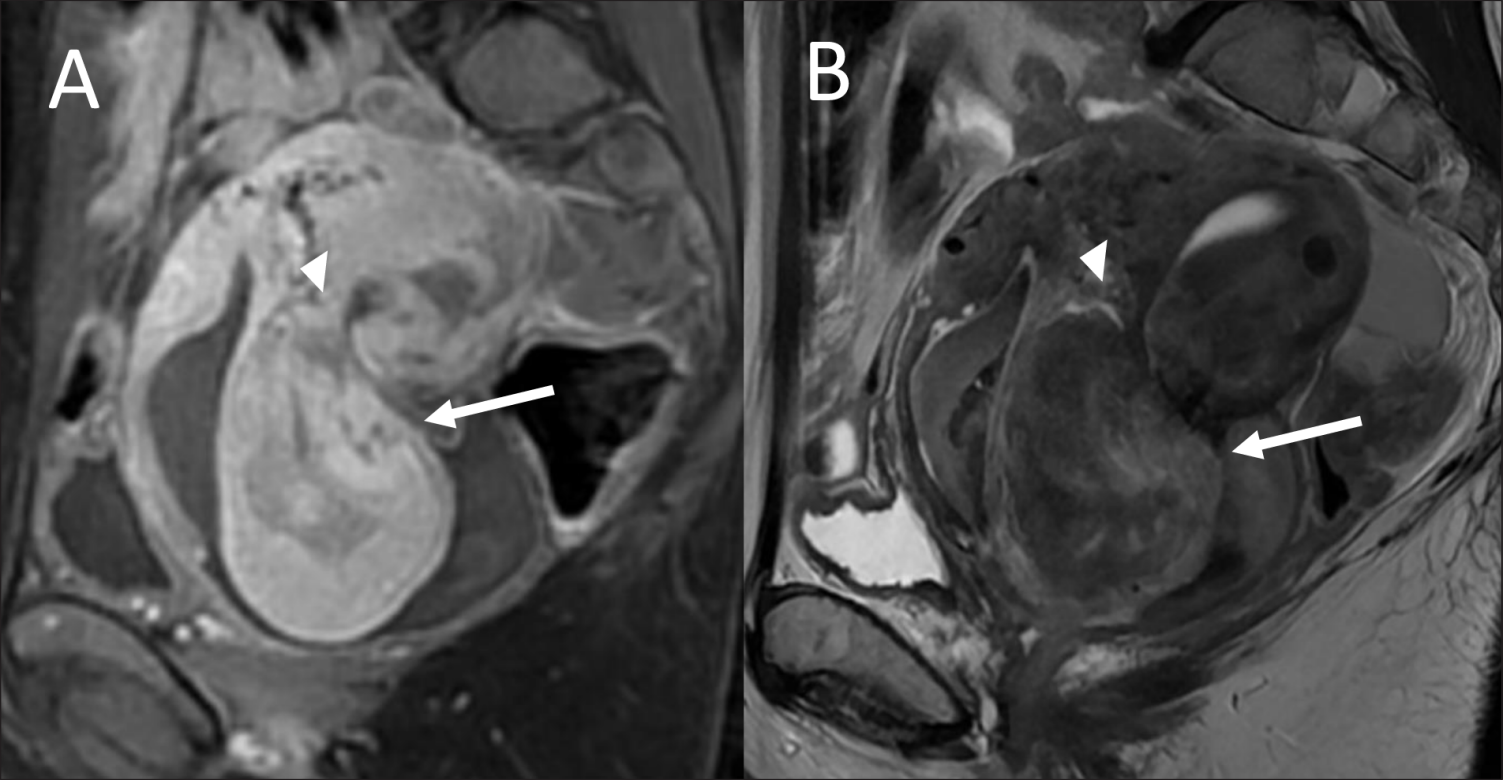


Sagittal gadolinium-enhanced fat-saturated T1-weighted image shows similar enhancement of the solid components of the mass and the myometrial tissue (Figure 3A). Sagittal gadolinium-enhanced fat-saturated T1-weighted image shows a very similar appearance between the solid component of the mass and myometrial tissue (Figure 3A, white arrow). This is connected to the endometrial cavity by a stalk (Figure 3A, 3B white head arrow).

The diagnosis of prolapsed uterine fibroid was confirmed by MRI and histopathology after surgery.

## Comments

Most PSUF are considered pedunculated fibroids classified FIGO 0. Their incidence is 2.5% and usual symptoms are vaginal bleeding, pelvic pain, cramping, or torsion. Diagnosis can be made with vaginal examination, ultrasonography, computed tomography scan, and MRI.

In case of acute gynecological bleeding, differential diagnostic between benign and malignant lesion and location of the lesion (endometrium, cervix, or vagina) are challenging with gynecological examination and transvaginal ultrasonography.

Magnetic resonance imaging enables an accurate diagnosis and prompt surgical treatment.

It reveals a mass prolapsed in the cervical and vaginal canal with a stalk attached to the myometrium of the uterine body. The combination of a stalk with prolapsed tumor and its enhancement similar to the myometrium’s enhancement is what is called the broccoli sign [1].
